# Circ_001569 regulates FLOT2 expression to promote the proliferation, migration, invasion and EMT of osteosarcoma cells through sponging miR-185-5p

**DOI:** 10.1515/biol-2020-0050

**Published:** 2020-07-10

**Authors:** Bin Xiao, Xusheng Zhang, Xiaojuan Li, Zhipeng Zhao

**Affiliations:** Department of Orthopaedic, Second People’s Hospital of Gansu Province, No. 1 Hezheng West Street, Chengguan District, Lanzhou City, Gansu Province, 730000, China; Department of General Surgery, Second People’s Hospital of Gansu Province, 730000, Lanzhou, Gansu, China; Department of Endocrine, Second People’s Hospital of Gansu Province, 730000, Lanzhou, Gansu, China

**Keywords:** osteosarcoma, circ_001569, miR-185-5p, FLOT2, proliferation, metastasis

## Abstract

Osteosarcoma (OS) is a common malignant tumor in the world. Circular RNAs are endogenous non-coding RNAs that have been linked to the development of cancer. However, the role of circ_001569 in OS progression is still unclear. Quantitative real-time polymerase chain reaction (qRT-PCR) was used to detect the expression of circ_001569, microRNA-185-5p (miR-185-5p) and flotillin-2 (FLOT2). The abilities of cell proliferation, migration and invasion were evaluated by the 3-(4,5-dimethyl-2-thiazolyl)-2,5-diphenyl-2-*H*-tetrazolium bromide (MTT) and Transwell assays, respectively. Also, western blot analysis was performed to assess the levels of epithelial–mesenchymal transition (EMT)-related proteins and FLOT2 protein. Besides, the dual-luciferase reporter assay was used to verify the interactions among circ_001569, miR-185-5p and FLOT2. Circ_001569 expression was increased in OS tissues and cells, and its knockdown reduced the proliferation, migration, invasion and EMT of OS cells. MiR-185-5p could interact with circ_001569. Inhibition of miR-185-5p could recover the suppression effects of silenced-circ_001569 on the proliferation and metastasis of OS cells. Furthermore, FLOT2 was a target of miR-185-5p. Overexpressed FLOT2 could restore the inhibition effects of miR-185-5p mimic on the proliferation and metastasis of OS cells. Also, FLOT2 expression was regulated by circ_001569 and miR-185-5p. In addition, circ_001569 knockdown also reduced the OS tumor growth *in vivo*. Circ_001569 might act as an oncogene in OS progression by regulating the miR-185-5p/FLOT2 axis, which provided a reliable new approach for the treatment of OS patients.

## 1 Introduction

Osteosarcoma (OS) is a malignant bone tumor originating from stromal cells and usually occurs in adolescents and children under 20 years of age [1,2]. Metastasis and recurrence are the leading causes of death in OS patients [3,4]. Although with the continuous improvement of early diagnosis and treatment techniques the prognosis of OS patients has been dramatically improved [5], the 5-year survival rate of OS patients with metastatic or multifocal diseases is still less than 25% [6]. Therefore, the exploration of new mechanisms affecting OS metastasis is expected to provide new ideas for reducing the death rate of OS.

Circular RNAs (circRNAs) are non-coding RNAs that do not encode proteins and are characterized by covalent closed-loop [7,8]. Many studies have confirmed that circRNAs play an active role in the development of various cancers, including hepatocellular carcinoma, breast cancer and glioblastoma [9,10,11]. In OS, Xi et al. identified a total of 259 differentially expressed circRNAs and found through gene ontology function enrichment analysis that they had significant enrichment in cell biological processes, cellular components and molecular functions [12]. This suggested that circRNA played an essential role in OS progression. Circ_001569 is a newly discovered circRNA and is located at chr16. In recent years, it had been found that high expression of circ_001569 was related to the progression of non-small cell lung cancer and hepatocellular carcinoma [13,14]. Zhang et al. reported that circ_001569 could promote OS cell proliferation and cisplatin resistance through the Wnt/β-catenin signaling pathway [15]. However, the effect of circ_001569 on the metastasis of OS and its molecular mechanism have not been investigated.

As small non-coding RNAs with a length of about 22 nucleotides, microRNAs (miRNAs) have been shown to be involved in the regulation of various cancers [16]. CircRNAs could bind to miRNAs as competitive endogenous RNA (ceRNA) and acted as miRNA sponges in cells, thus removing the suppression of miRNA on target genes to regulate its expression [17,18]. Research had shown that miR-185-5p took part in regulating the progression of many cancers, including hepatocellular carcinoma, glioma and melanoma [19,20,21]. Also, flotillin-2 (FLOT2), a member of the flotillin family, has been shown to be upregulated in many cancers and is associated with cancer progression [22,23,24]. However, their roles in OS have not been reported.

The goal of this study is to evaluate the biological function of circ_001569 in OS and determine its molecular mechanism through bioinformatics prediction and experimental verification. The discovery of circ_001569/miR-185-5p/FLOT2 axis perfected the role of circ_001569 as ceRNA, providing new thinking for targeted therapy in OS patients.

## 2 Materials and methods

### 2.1 Patients and sample collection

In this study, 30 OS patients were recruited from Second People’s Hospital of Gansu Province. OS tissues and adjacent normal tissues were taken and stored at −80°C until use. All patients did not receive any treatment. The clinicopathological information of OS patients was presented in Table 1.

**Table 1:** Clinicopathological information of OS patients

Clinicopathological parameters	Case (*n* = 30)
Age (years)	
≤25	16
>25	14
Gender	
Male	13
Female	17
TNM stage	
I–II	19
III–IV	11
Distant metastasis	
Absent	13
Present	17


**Informed consent:** Informed consent has been obtained from all individuals included in this study.
**Ethical approval:** The research related to human use has been complied with all the relevant national regulations, institutional policies and in accordance with the tenets of the Helsinki Declaration and has been approved by the Ethics Committee of Second People’s Hospital of Gansu Province.

### 2.2 Cell culture and transfection

OS cells (SaOS-2, U2OS, MG-63 and HOS) and human normal osteoblastic cells (hFOB) were bought from American Type Culture Collection (ATCC, Manassas, VA, USA). All cells were cultured in Dulbecco’s modified Eagle’s medium (DMEM; Gibco, Waltham, MA, USA) containing 10% fetal bovine serum (FBS; Gibco), 100 µg/mL of streptomycin (Gibco) and 100 U/mL of penicillin (Gibco) at 37°C with 5% CO_2_. Small interfering RNA (siRNA) against circ_001569 (si-circ_001569#1: 5′-CACUUUCUACGUCUACUUUUC-3′ and si-circ_001569#2: 5′-CCUUCUACUUCCUCUAUCUCU-3′) or its negative control (si-NC: 3′-UCACCCAGAUGCCGCUAU-5′), miR-185-5p mimic and inhibitor (miR-185-5p: F 5′-UGGAGAGAAAGGCAGUUCCUGA-3′, R 5′-AGGAACUGCCUUUCUCUCCAUU-3′; anti-miR-185-5p: 5′-UCAGGAACUGCCUUUCUCUCCA-3′) or their negative controls (miR-NC: 5′-CUAGUCAUCGAUGUCGUAGCA-3′ and anti-miR-NC: 5′-CAGUACAUUGGUUCUGCAA-3′), circ_001569 and FLOT2 overexpression plasmid (circ_001569 and FLOT2) or their negative control (vector) were purchased from RiboBio (Guangzhou, China). U2OS and HOS cells were seeded into six-well plates (1 × 10^6^ per well). When the cells reached 50–60% confluence, cell transfection could be performed. The cells were transfected with all plasmids and oligonucleotides at a final concentration of 20 nM using 5 µL of Lipofectamine 3000 (Invitrogen, Carlsbad, CA, USA) according to the manufacturer’s protocol.

### 2.3 Quantitative real-time polymerase chain reaction (qRT-PCR)

Cells and tissues were lysed with TRIzol reagent (Invitrogen) for RNA extraction. TaqMan Reverse Transcription Reagent (Invitrogen) was used to reverse transcribe RNA into cDNA. SYBR Green (Solarbio, Beijing, China) was used for qRT-PCR on an ABI 7500 System (Applied Biosystems, Foster City, CA, USA). The relative expression was normalized to glyceraldehyde 3-phosphate dehydrogenase (GAPDH) or U6 and analyzed using the 2^−ΔΔCt^ method. The primers were as follows: circ_001569: F 5′-TCCCCTGAACATTCTCCCCAT-3′, R 5′-GAAAGCACTTGGTGAAGTCGG-3′; FLOT2: F 5′-CCCCAGATTGCTGCCAAA-3′, R 5′-TCCACTGAGGACCACAATCTCA-3′; GAPDH: F 5′-ACCACAGTCCATGCCATCAC-3′, R 5′-TCCACCACCCTGTTGCTGTA-3′; miR-185-5p: F 5′-GAAGGATCCGCATGAGAGGGTGTTGGAATGC-3′, R 5′-GGAGAATTCGTGCAGGGGCAGCAGACC-3′; and U6: F 5′-GCAGGAGGTCTTCACAGAGT-3′, R 5′-TCTAGAGGAGAAGCTGGGGT-3′.

### 2.4 Cell proliferation assay

U2OS and HOS cells were seeded into 96-well plates. The ability of OS cell proliferation was detected using 3-(4,5-dimethyl-2-thiazolyl)-2, 5-diphenyl-2-*H*-tetrazolium bromide (MTT) Assay Kit (Beyotime, Shanghai, China) at the specified time point according to the manufacturer’s protocol.

### 2.5 Transwell assay

Cell migration and invasion assays were performed using chambers with 8 µm pore size polycarbonate membranes (Corning Inc., Corning, NY, USA). Chambers coated with Matrigel (BD Biosciences, Franklin, NJ, USA) were used to detect cell invasion and uncoated chambers were used to detect cell migration. Serum-free DMEM was filled into the upper chambers, and DMEM containing 10% FBS was filled into the lower chambers. After 24 h, cells on the surface of lower chambers were fixed and stained. Then, cells were photographed and counted using a microscope (Shoif, Shanghai, China).

### 2.6 Western blot (WB) analysis

Protein extraction was performed using lysis buffer (Beyotime). Equal amounts of proteins were isolated with 10% sodium dodecyl sulfate-polyacrylamide gel electrophoresis gel and transferred onto polyvinylidene difluoride membranes (Millipore, Billerica, MA, USA). After blockage with 5% fat-free milk, membranes were incubated with primary antibodies against *N*-cadherin (1:1,500; GeneTex, Irvine, CA, USA), *E*-cadherin (1:3,000; GeneTex), Vimentin (1:5,000; GeneTex), GAPDH (1:5,000; GeneTex), FLOT2 (1:1,000; GeneTex) at 4°C overnight, and then incubated with horseradish peroxidase-conjugated secondary antibodies (1:2,000; GeneTex) for 1 h. Protein levels were measured by enhanced chemiluminescence solution (Beyotime).

### 2.7 Dual-luciferase reporter assay

The recombinant reporter vectors of circ_001569 WT/MUT and FLOT2 3′-UTR-WT/MUT were built using pmirGLO vectors (Promega, Madison, WI, USA). U2OS and HOS cells were seeded into the six-well culture plates. The aforementioned vectors were cotransfected with miR-185-5p mimic or miR-NC into cells. Luciferase activity was measured using the Dual-Luciferase Assay System (Promega) after transfection of 48 h.

### 2.8 OS tumor xenograft model

HOS cells (2 × 10^6^/0.2 mL PBS) transfected with lentiviral short hairpin RNA against circ_001569 or its negative control (sh-circ_001569 or sh-NC) were injected subcutaneously into the flank of BALB/c nude mice (Guangdong Medical Laboratory Animal Center, Guangdong, China). Tumor length and width were measured weekly with calipers, and tumor volume was calculated using the formula: length × width^2^/2. After 5 weeks, the mice were euthanized, the tumor was taken out and weighed. QRT-PCR or WB analysis was used to detect the expression of circ_001569, miR-185-5p and FLOT2 in the tumor tissues.


**Ethical approval:** The research related to animal use has been complied with all the relevant national regulations and institutional policies for the care and use of animals and has been approved by the Animal Ethics Committee of Second People’s Hospital of Gansu Province.

### 2.9 Statistical analysis

Data were presented as mean ± standard deviation. The statistical analysis was carried out using GraphPad Prism 6 (GraphPad Software Inc., San Diego, CA, USA). Student’s *t*-test and one-way analysis of variance were used to determine statistical significance. *P* < 0.05 was considered as statistically significant.

## 3 Results

### 3.1 Circ_001569 was upregulated in OS tissues and cells

**Figure 1:**
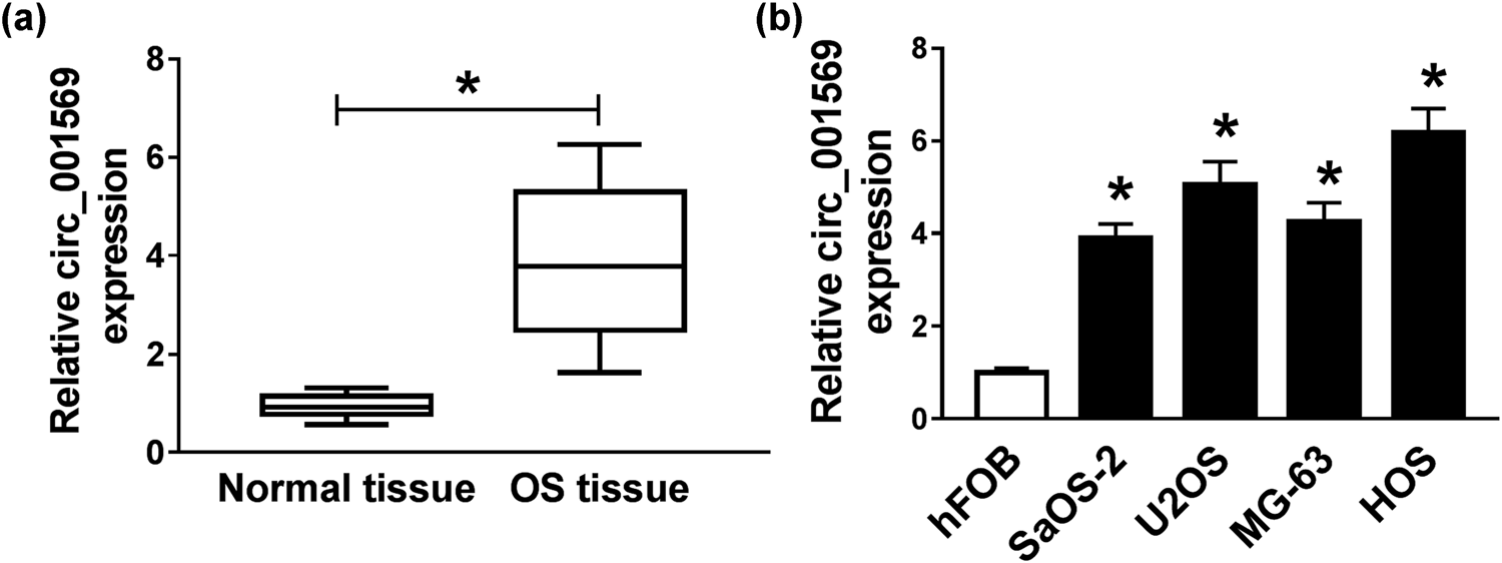
The expression of circ_001569 in OS. (a) The expression of circ_001569 was detected by qRT-PCR in OS tissues and adjacent normal tissues. (b) QRT-PCR was used to measure circ_001569 expression in OS cells (SaOS-2, U2OS, MG-63 and HOS) and human normal osteoblastic cells (hFOB). *P < 0.05.

To evaluate the role of circ_001569 in OS development, we detected the expression level of circ_001569 in 30 pairs of OS tissues and adjacent normal tissues using qRT-PCR. Our results revealed that circ_001569 expression was significantly higher in OS tissues (*P* < 0.0001; Figure 1a). Furthermore, it was also observed that the expression of circ_001569 was dramatically increased in four OS cells (SaOS-2, U2OS, MG-63 and HOS), especially in U2OS and HOS cells, when compared with that of the human normal osteoblastic cells (hFOB; *P* < 0.0001; Figure 1b). These data suggested that circ_001569 might be involved in the development of OS.

### 3.2 Inhibition of circ_001569 suppressed the proliferation, migration, invasion and epithelial–mesenchymal transition (EMT) of OS cells

**Figure 2:**
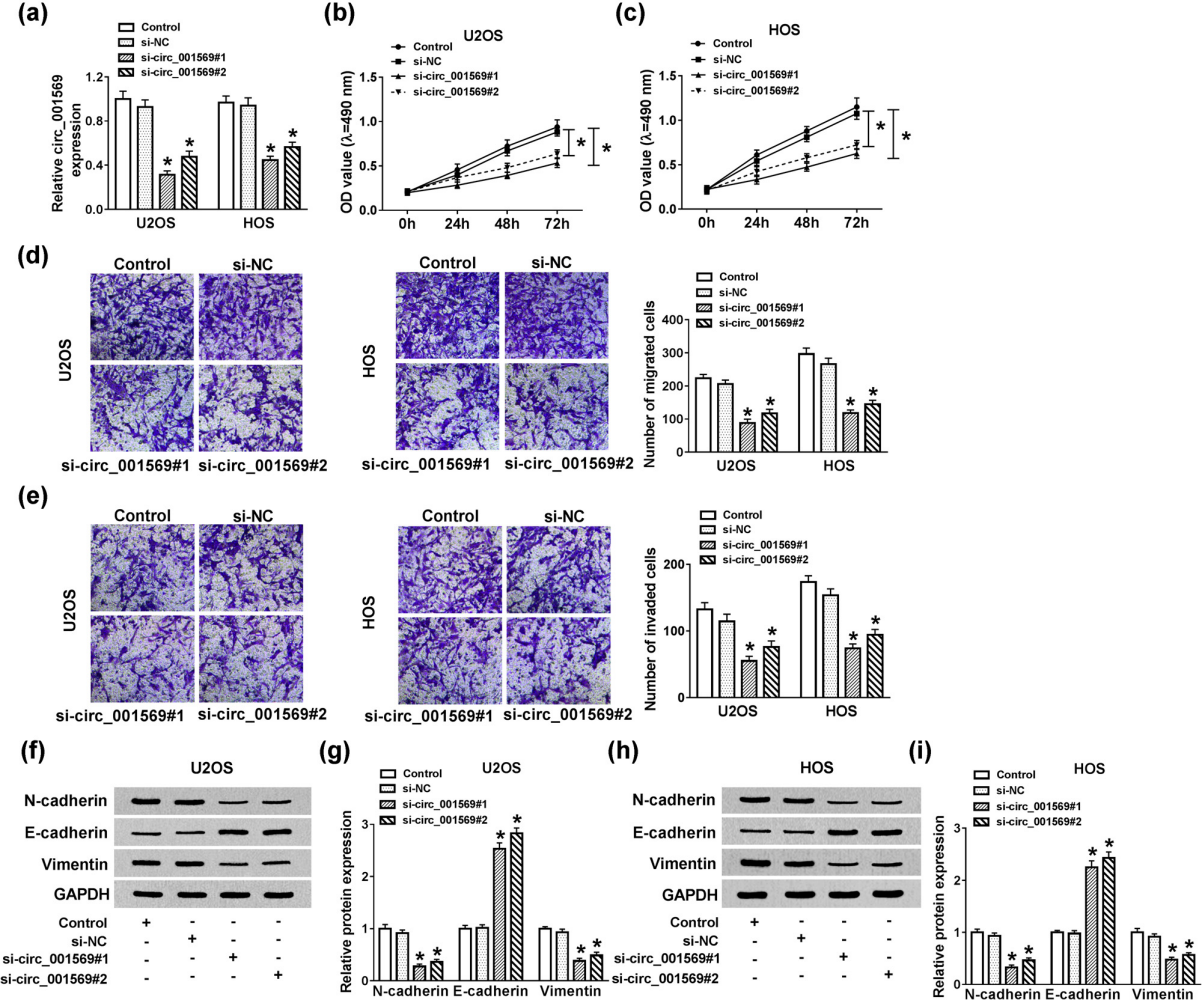
Effects of circ_001569 knockdown on the progression of OS cells. U2OS and HOS cells were transfected with si-circ_001569#1/#2 or si-NC. (a) The expression of circ_001569 was detected by qRT-PCR to evaluate the transfection efficiency of si-circ_001569#1/#2. (b and c) MTT assay was performed to assess the proliferation ability of U2OS and HOS cells. (d and e) Transwell assay was performed to detect the migration and invasion of U2OS and HOS cells. (f–i) The levels of EMT-related proteins (*N*-cadherin, *E*-cadherin and Vimentin) in U2OS and HOS cells were detected by WB analysis. *P < 0.05.

To explore the biological function of circ_001569 in OS, we transfected si-circ_001569#1/#2 into U2OS and HOS cells to measure the effect of circ_001569 silencing on OS cell progression. The efficacy of si-circ_001569#1/#2 transfection was assessed by qRT-PCR. It was found that the circ_001569 level in the si-circ_001569#1/#2 group was significantly lower than that in the si-NC group (*P* < 0.0001; Figure 2a). The MTT assay was used to assess the proliferation of OS cells. As shown in Figure 2b and c, knockdown of circ_001569 markedly repressed the proliferation of U2OS and HOS cells (*P* < 0.001). Besides, Transwell assay was performed to explore the effects of silenced-circ_001569 on the metastasis of OS cells. As the results presented in Figure 2d and e, inhibition of circ_001569 impeded the number of migrated and invaded cells of OS (*P* < 0.001), suggesting that circ_001569 could enhance the migration and invasion of OS cells. Also, we further conducted WB analysis to evaluate the changes of EMT-related proteins in OS cells. The results demonstrated that inhibition of circ_001569 markedly reduced the protein levels of *N*-cadherin and Vimentin, while increased the *E*-cadherin protein level in U2OS and HOS cells (*P* < 0.0001; Figure 2f–i). Overall, these data indicated a promoting role of circ_001569 in the proliferation and metastasis of OS cells.

### 3.3 Circ_001569 directly targeted miR-185-5p

**Figure 3:**
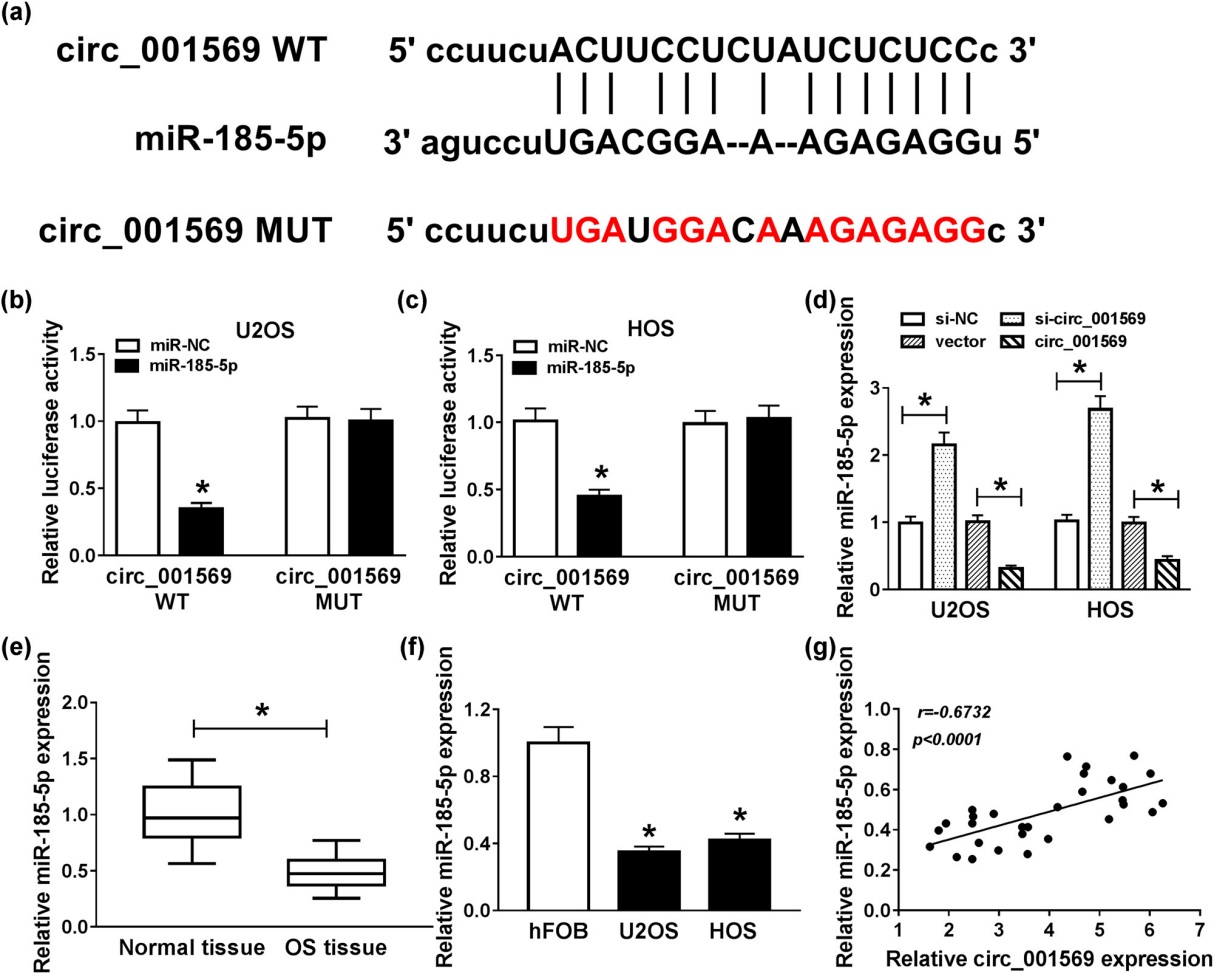
Circ_001569 sponged miR-185-5p. (a) Circ_001569 containing miR-185-5p binding sites (circ_001569 WT) and mutant binding sites (circ_001569 MUT) are shown. (b and c) Dual-luciferase reporter assay was used to detect the interaction between miR-185-5p and circ_001569 in U2OS and HOS cells. (d) The effect of circ_001569 expression on the expression of miR-185-5p in U2OS and HOS cells was detected by qRT-PCR. (e and f) The expression of miR-185-5p was lower in OS tissues and cells detected by qRT-PCR. (g) The correlation between miR-185-5p and circ_001569 expression was measured by Pearson correlation coefficient analysis. *P < 0.05.

To explore that the mechanism of circ_001569 regulated the progression of OS cells, we used the StarBase v2.0 tools to predict the target of circ_001569 and found that miR-185-5p had a complementary binding site with circ_001569 (Figure 3a). Through the dual-luciferase reporter assay, we found that miR-185-5p overexpression remarkably reduced the luciferase activity of circ_001569 WT in U2OS and HOS cells (*P* < 0.0001), but not that of circ_001569 MUT (Figure 3b and c). Besides, we also discovered that knockdown of circ_001569 improved miR-185-5p expression, while overexpression of circ_001569 inhibited its expression (*P* < 0.001; Figure 3d). QRT-PCR results indicated that the expression of miR-185-5p was lower in OS tissues and cells (*P* < 0.0001; Figure 3e and f). Moreover, correlation analysis revealed that the expression of miR-185-5p was negatively correlated with circ_001569 in OS tissues (*P* < 0.0001; Figure 3g). Therefore, these observations demonstrated that miR-185-5p could interact with circ_001569 in OS cells.

### 3.4 Silenced-miR-185-5p partially reversed the effects of circ_001569 knockdown on OS cell progression

**Figure 4:**
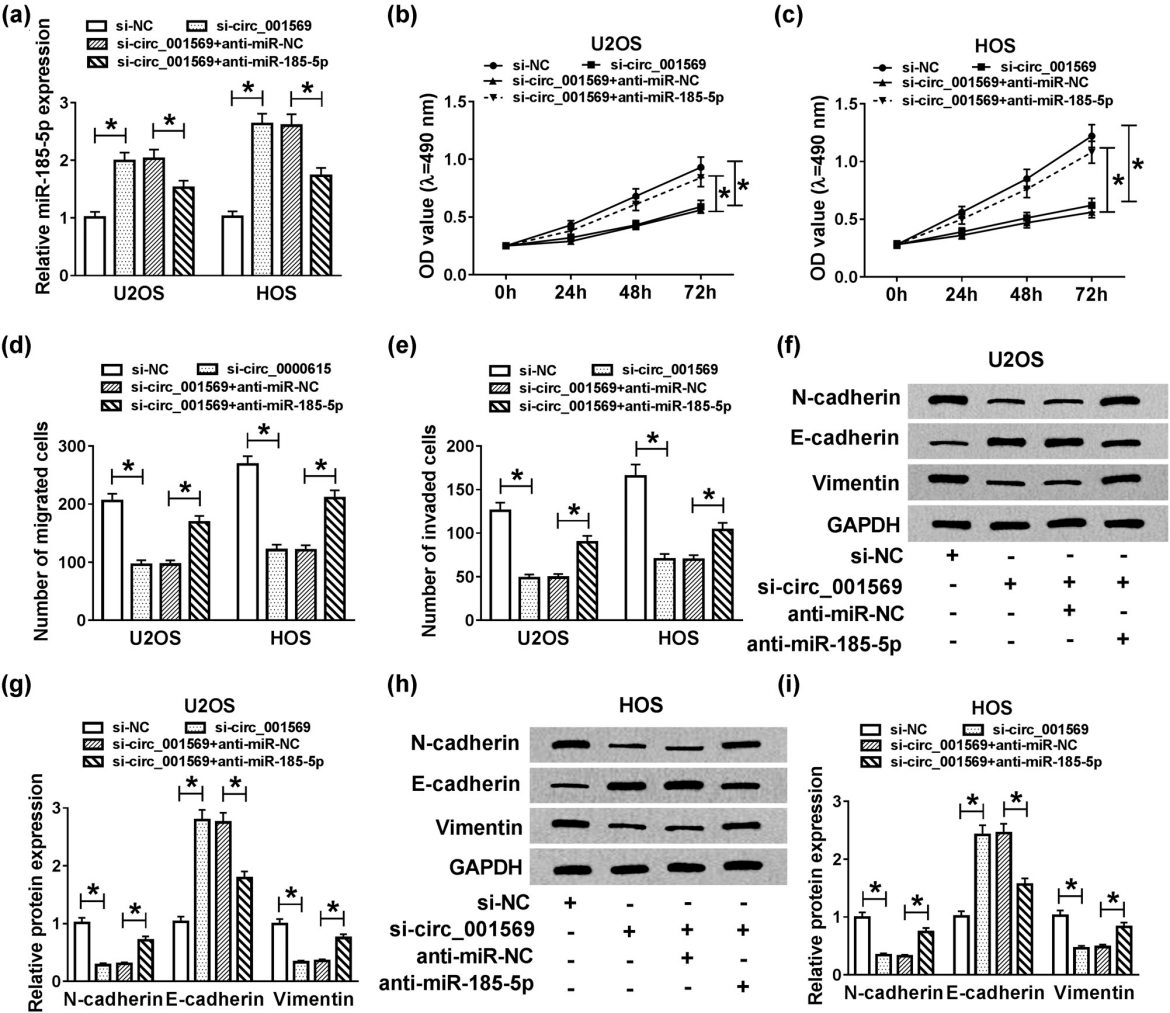
Effects of miR-185-5p inhibitor and circ_001569 knockdown on the progression of OS cells. U2OS and HOS cells were cotransfected with si-circ_001569 and anti-miR-185-5p or their negative controls (si-NC and anti-miR-NC). (a) The expression of miR-185-5p was detected by qRT-PCR to evaluate the transfection efficiency of si-circ_001569 and anti-miR-185-5p. (b and c) The proliferation of U2OS and HOS cells was detected by the MTT assay. (d and e) The migration and invasion of U2OS and HOS cells were measured by the Transwell assay. (f–i) The protein levels of *N*-cadherin, *E*-cadherin and Vimentin in U2OS and HOS cells were detected by WB analysis. *P < 0.05.

To evaluate whether circ_001569 affected OS cell progression through miR-185-5p, si-circ_001569 and anti-miR-185-5p were cotransfected into U2OS and HOS cells to investigate the role of miR-185-5p. The transfection efficiency was reflected by the detection of miR-185-5p expression using qRT-PCR, and the results indicated that the transfection efficiency of si-circ_001569 and anti-miR-185-5p was excellent (*P* < 0.01; Figure 4a). MTT and Transwell assay results showed that inhibition of miR-185-5p could restore the suppression influences of proliferation, migration and invasion on U2OS and HOS cells after circ_001569 knockdown (*P* < 0.05; Figure 4b–e). Furthermore, miR-185-5p inhibitor also restored the promotion of silenced-circ_001569 on the protein level of *E*-cadherin and the inhibition of it on the protein levels of *N*-cadherin and Vimentin (*P* < 0.001; Figure 4f–i), indicating that miR-185-5p inhibitor promoted the EMT process. Hence, these results suggested that miR-185-5p played a vital role in the regulation of OS progression by circ_001569.

### 3.5 FLOT2 was a target of miR-185-5p

**Figure 5:**
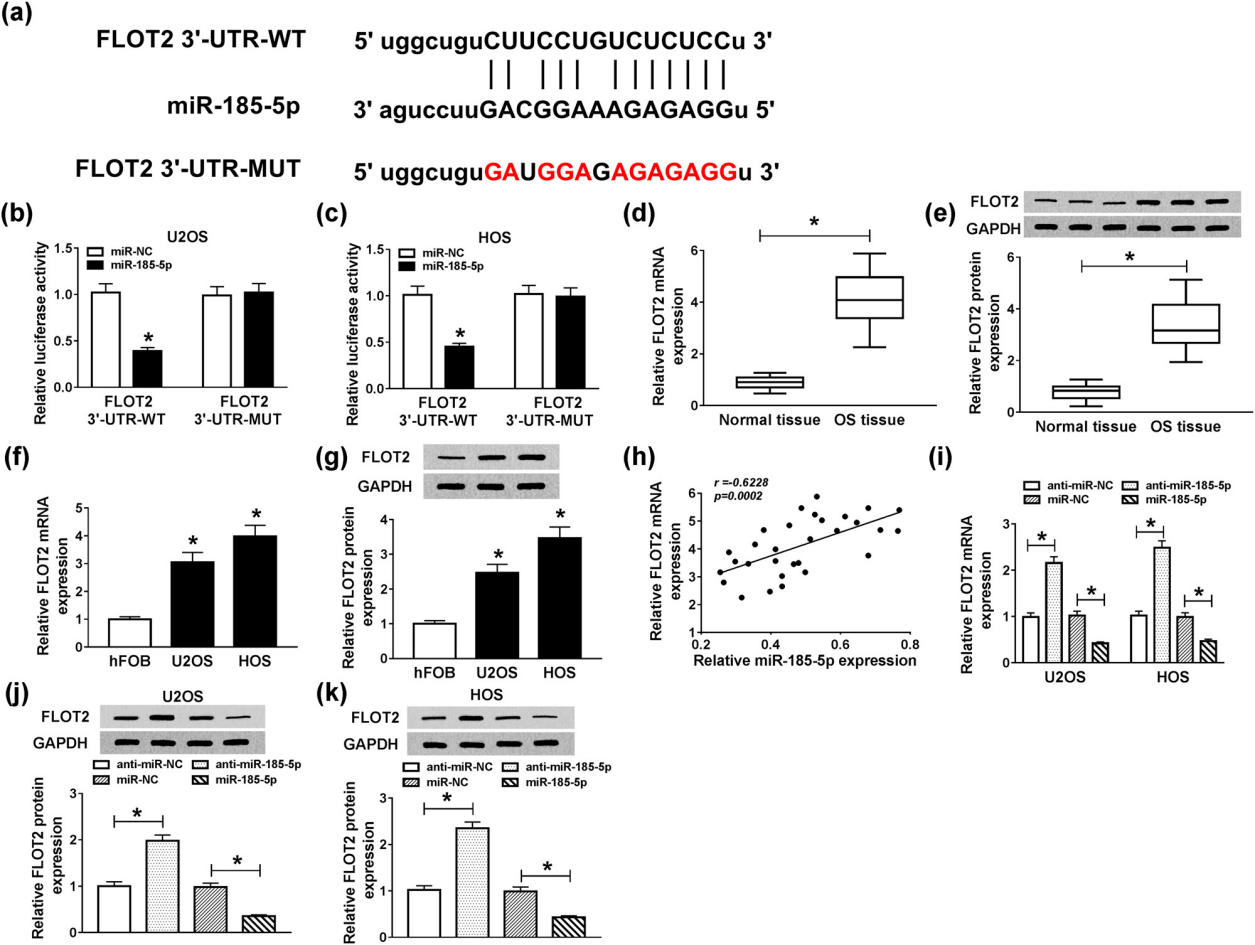
FLOT2 was directly targeted by miR-185-5p. (a) The 3′-UTR of FLOT2 containing miR-185-5p binding sites (FLOT2 3′-UTR-WT) and mutant binding sites (FLOT2 3′-UTR-WT) were shown. (b and c) Dual-luciferase reporter assay was used to detect the interaction between miR-185-5p and FLOT2 in U2OS and HOS cells. (d and e) The expression of FLOT2 in OS tissues and adjacent normal tissues was determined by qRT-PCR and WB analysis. (f and g) QRT-PCR and WB analyses were performed to detect the expression of FLOT2 in OS cells (SaOS-2, U2OS, MG-63 and HOS) and human normal osteoblastic cells (hFOB). (h) The correlation between FLOT2 and miR-185-5p expression was evaluated by Pearson correlation coefficient analysis. (i–k) The effect of miR-185-5p expression on the level of FLOT2 in U2OS and HOS cells was detected by qRT-PCR and WB analyses. *P < 0.05.

With the help of the StarBase v2.0 tools, we found a putative miR-185-5p binding site located in the 3′-UTR of FLOT2 (Figure 5a). To confirm whether miR-185-5p directly bonded to FLOT2, we cloned FLOT2 3′-UTR-WT/MUT to perform the dual-luciferase reporter assay. The results revealed that miR-185-5p overexpression markedly inhibited the luciferase activity of FLOT2 3′-UTR-WT in U2OS and HOS cells (*P* < 0.0001), while did not affect FLOT2 3′-UTR-MUT (Figure 5b and c). Through the detection of mRNA and protein expression levels, we found that FLOT2 was highly expressed in OS tissues and cells (*P* < 0.001; Figure 5d–g). Meanwhile, we performed correlation analysis and found that FLOT2 expression was negatively correlated with miR-185-5p (*P* = 0.0002; Figure 5h). Also, we investigated the effect of miR-185-5p expression on FLOT2 expression in U2OS and HOS cells and discovered that knockdown of miR-145-5p promoted the expression of FLOT2, while overexpression of miR-145a-5p hindered its expression (*P* < 0.0001; Figure 5i). Similarly, we also found the same results at the protein level (*P* < 0.001, Figure 5j and k). These results revealed that miR-185-5p targeted FLOT2 in OS cells.

### 3.6 Overexpressed-FLOT2 partially inversed the effects of miR-185-5p aberrant expression on OS cell progression

**Figure 6:**
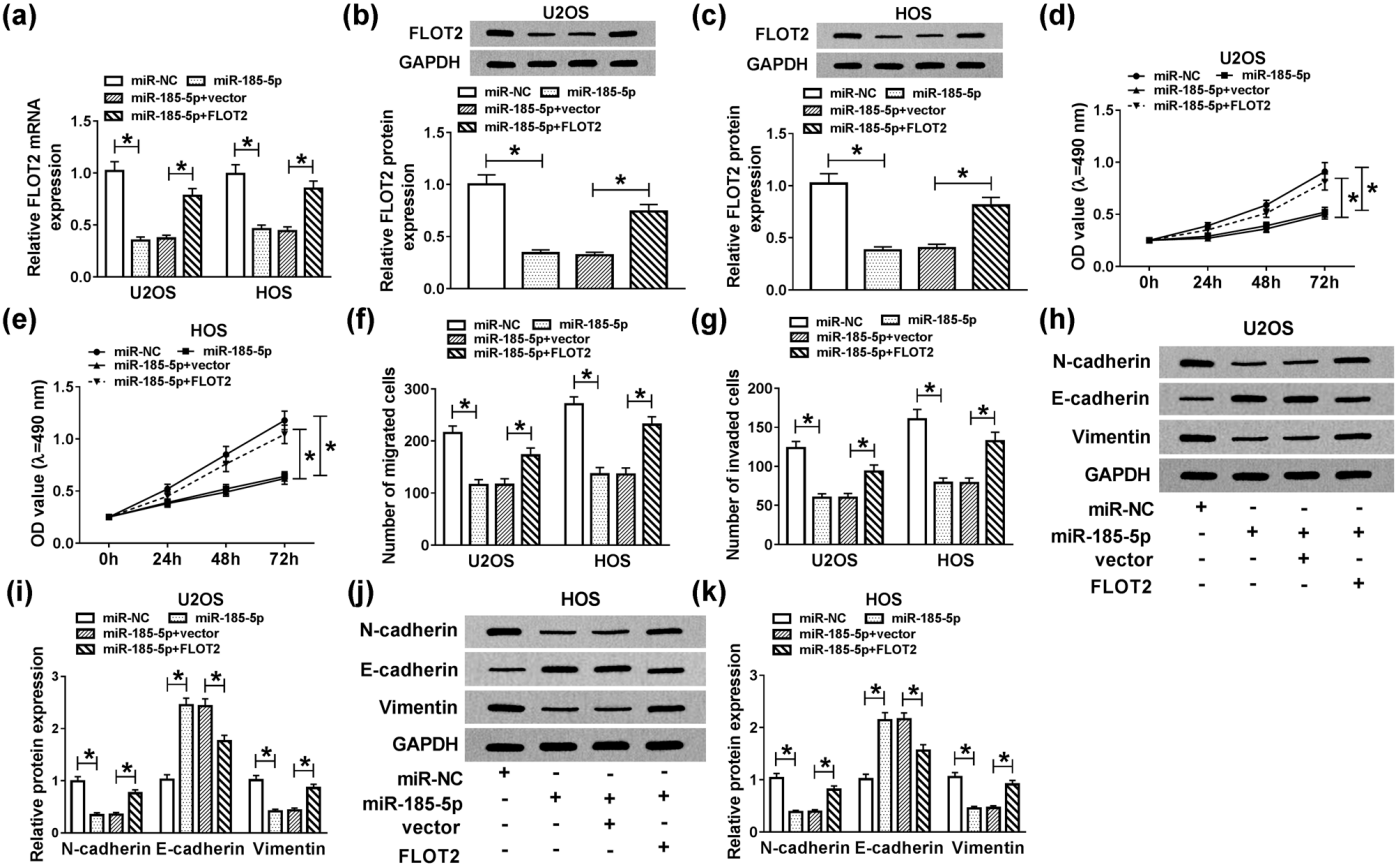
Effects of FLOT2 and miR-185-5p overexpression on the progression of OS cells. U2OS and HOS cells were cotransfected with miR-185-5p mimic and FLOT2 overexpression plasmid or their negative controls (miR-NC and vector). (a–c) The expression of FLOT2 was detected by qRT-PCR and WB analysis to evaluate the transfection efficiency. (d and e) The proliferation was detected by the MTT assay in U2OS and HOS cells. (f and g) The migration and invasion were measured by the Transwell assay in U2OS and HOS cells. (h–k) The protein levels of *N*-cadherin, *E*-cadherin and Vimentin were detected by WB analysis in U2OS and HOS cells. *P < 0.05.

To make clear the influence of interaction between miR-185-5p and FLOT2 on OS cell progression, miR-185-5p mimic and FLOT2 overexpression plasmid were cotransfected into U2OS and HOS cells. QRT-PCR and WB analysis results revealed that the level of FLOT2 was suppressed by miR-185-5p upregulation in U2OS and HOS cells, while was promoted by the introduction of FLOT2 (*P* < 0.001; Figure 6a–c). MTT and Transwell assay results indicated that the abnormally expressed miR-185-5p decreased the proliferation, migration and invasion of U2OS and HOS cells. On the contrary, FLOT2 overexpression restored the function of miR-185-5p, promoting the proliferation and metastasis of OS cells (*P* < 0.05; Figure 6d–g). Then, *N*-cadherin, *E*-cadherin and Vimentin levels were detected in U2OS and HOS cells, and the results disclosed that miR-185-5p repressed the expression of *N*-cadherin and Vimentin and enhanced *E*-cadherin level, while FLOT2 overexpression could reverse the effect of miR-185-5p (*P* < 0.0001; Figure 6h–k). Thus, FLOT2 might act as an oncogene in OS cells.

### 3.7 Circ_001569 regulated FLOT2 expression through sponging miR-185-5p

**Figure 7:**
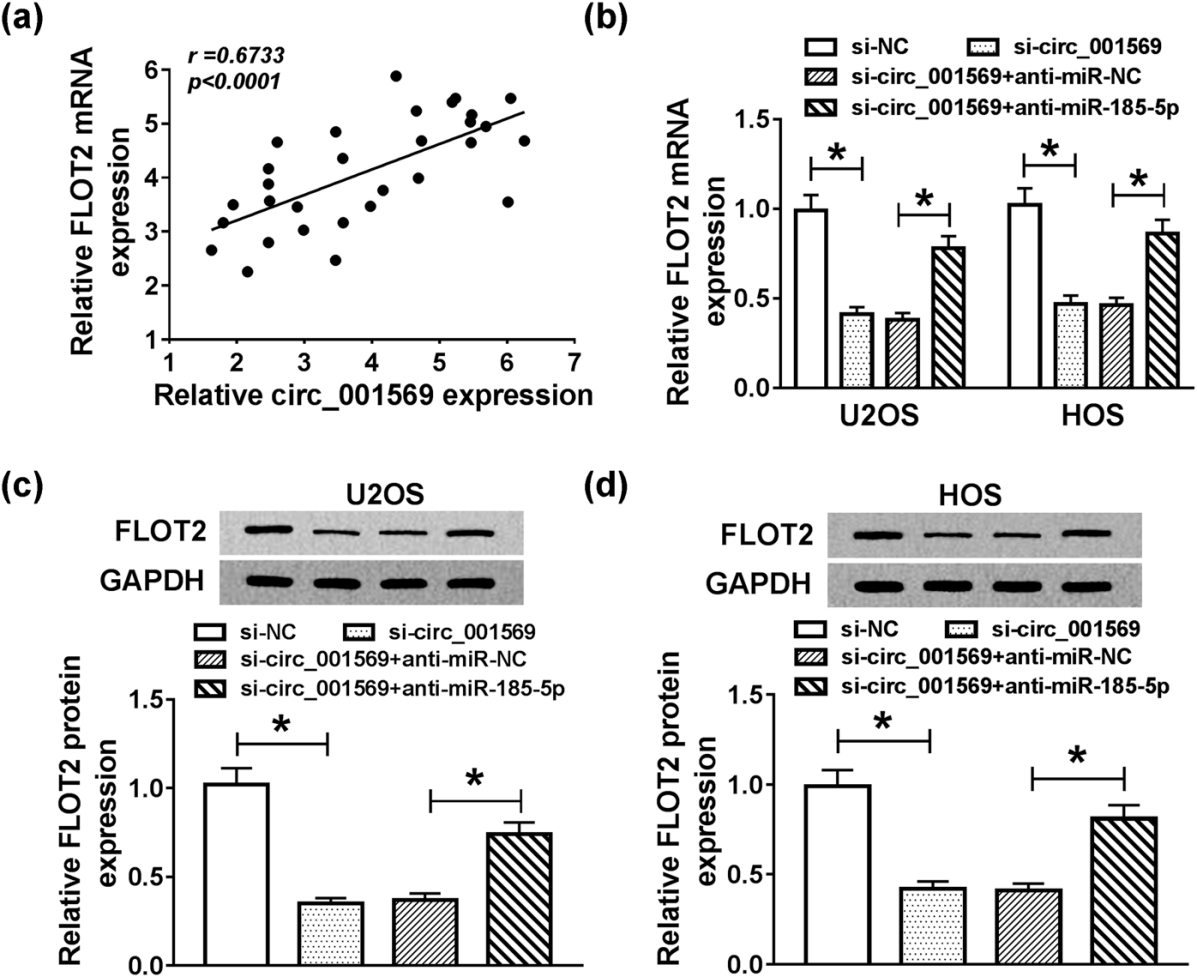
Effects of circ_001569 and miR-185-5p knockdown on the expression of FLOT2. (a) Pearson correlation coefficient analysis was performed to assess the correlation between FLOT2 and circ_001569 expression. U2OS and HOS cells were cotransfected with si-circ_001569 and anti-miR-185-5p or their negative controls (si-NC and anti-miR-NC). (b) The expression of FLOT2 in U2OS and HOS cells was detected by qRT-PCR. (c and d) WB analysis was used to evaluate the protein level of FLOT2 in U2OS and HOS cells. *P < 0.05.

To determine the regulatory relationship between circ_001569 and FLOT2, we conducted a correlation analysis and found that their expression levels were significantly positively correlated (*P* < 0.0001; Figure 7a). Meanwhile, we detected the expression of FLOT2 in mRNA and protein levels, and the results indicated that silenced-circ_001569 inhibited the expression of FLOT2 in U2OS and HOS cells, while interfering of miR-185-5p could improve its expression (*P* < 0.001; Figure 7b–d). These data revealed that circ_001569 regulated FLOT2 expression through sponging miR-185-5p in OS cells.

### 3.8 Circ_001569 knockdown reduced OS tumor growth *in vivo*

**Figure 8:**
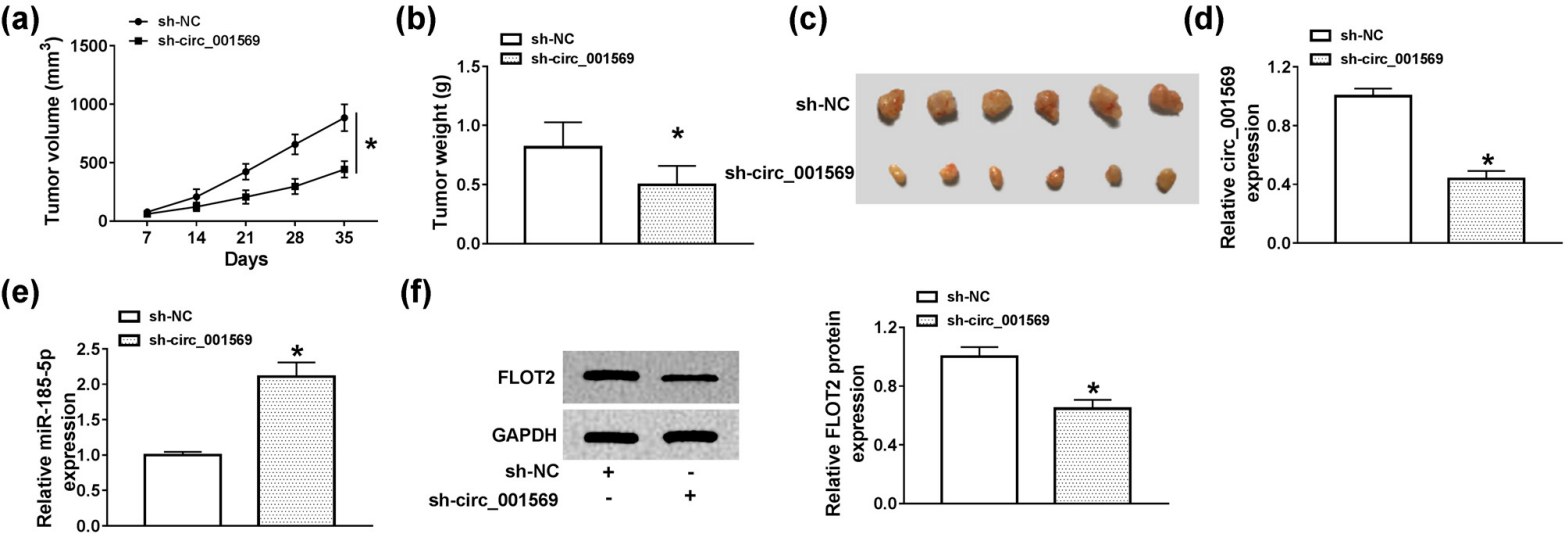
Circ_001569 knockdown reduced OS tumor growth *in vivo*. HOS cells transfected with sh-NC or sh-circ_001569 were injected into nude mice. The tumor volume (a) and tumor weight (b) were measured in mice. (c) The pictures of tumor tissue in each group. (d and e) QRT-PCR was used to detect the expression of circ_001568 and miR-185-5p in the tumor tissues. (f) The protein expression of FLOT2 was determined by WB analysis. *P < 0.05.

To further evaluate the role of circ_001569 in OS, we constructed OS tumor xenograft models. We found that compared with the sh-NC group, the growth rate of tumor volume in the sh-circ_001569 group was significantly reduced (*P* < 0.0001; Figure 8a). By detecting tumor weight, we determined that the tumor weight of the sh-circ_001569 group was markedly smaller than in the sh-NC group (*P* < 0.05; Figure 8b and c). In order to confirm the success of the knockdown of circ_001569 in the sh-circ_001569 group, we tested the expression of circ_001569 in the tumor tissue, and the results showed that the expression of circ_001569 in the sh-circ_001569 group was remarkably lower than that of the sh-NC group, indicating that the transfection of sh-circ_001569 was successful (*P* < 0.001; Figure 8d). In addition, we also discovered that miR-185-5p expression was enhanced and FLOT2 protein expression was inhibited in the sh-circ_001569 group (*P* < 0.01, Figure 8e and f). These results revealed that circ_001569 silencing could inhibit the tumor growth of OS by regulating the miR-185-5p/FLOT2 axis.

## 4 Discussion

At present, with the deepening of research, it has been found that circRNAs have potential functions in cancers [25]. As a critical regulatory factor in biological processes, circRNAs can affect tumor proliferation and metastasis and may also become a potential diagnostic biomarker or therapeutic target for cancers [26,27]. For example, circSAMD4A promoted cell proliferation by regulating the miR-1244/MDM2 axis [28]. Also, circTADA2A improved CREB3 expression to enhance OS progression and metastasis by sponging miR-203a-3p and functioned as a tumor promoter in OS [29]. Besides, circ_0000502 could sponge miR-1238 to facilitate OS progression and act as an unfavorable prognosis indicator in OS [30]. Here, we found that circ_001569 was abnormally expressed in OS. Knockdown of circ_001569 remarkably suppressed the proliferation, migration, invasion and EMT of OS cells *in vitro* and reduced the tumor growth of OS *in vivo*. Our results suggested that circ_001569 played an essential role in the development of OS.

At present, many miRNAs were involved in the regulation of OS. Xu et al. reported that miR-106b was associated with poor prognosis for OS patients and regulated the progression of OS [31]. Moreover, Zhao et al. showed that miR-495-3p could regulate the expression of CTRP3 to suppress the proliferation and metastasis of OS [32]. Here, we used bioinformatics analysis and further experimental verification to find that circ_001569 directly sponged miR-185-5p. Previous studies had shown that the expression of miR-185-5p was lower in cancers [19,20]. In our research, we discovered that miR-185-5p expression was decreased in OS and was regulated by circ_001569 expression. Furthermore, miR-185-5p inhibitor partially inverted the suppression effects of circ_001569 silencing on the proliferation and metastasis of OS cells. On the contrary, overexpression of miR-185-5p hindered OS cell progression. Therefore, we indicated that circ_001569 exerted an oncogenic function through sponging miR-185-5p in OS cells.

Using the same prediction and validation methods, we showed that FLOT2 was a target of miR-185-5p. Wang et al. reported that FLOT2 targeted by miR-34c-5p to participate in the development of OS [33]. Consistent with previous studies, this study verified that FLOT2 expression was increased in OS tissues and cells. Also, upregulated FLOT2 promoted the proliferation and metastasis of OS cells and reversed the suppression effects of miR-185-5p overexpression on OS progression. In addition, FLOT2 expression was regulated by the expression levels of circ_001569 and miR-185-5p, which reduced with the knockdown of circ_001569 and increased with the inhibition of miR-185-5p. Hence, we demonstrated that circ_001569 promoted OS cell progression by increasing FLOT2 expression through targeting miR-185-5p in OS.

In conclusion, we confirmed that circ_001569 absorbed miR-185-5p to improve FLOT2 expression, thereby promoting the proliferation and metastasis of OS cells, which exerted an oncogenic role in OS. The elucidation of circ_001569 molecular mechanism revealed that it could be a therapeutic target for OS.
